# Type I Interferon Signaling Regulates Ly6C^hi^ Monocytes and Neutrophils during Acute Viral Pneumonia in Mice

**DOI:** 10.1371/journal.ppat.1001304

**Published:** 2011-02-24

**Authors:** Sang-Uk Seo, Hyung-Joon Kwon, Hyun-Jeong Ko, Young-Ho Byun, Baik Lin Seong, Satoshi Uematsu, Shizuo Akira, Mi-Na Kweon

**Affiliations:** 1 Mucosal Immunology Section, International Vaccine Institute, Seoul, South Korea; 2 Laboratory of Microbiology and Immunology, College of Pharmacy, Kangwon National University, Chuncheon, South Korea; 3 Department of Biotechnology, College of Engineering, Yonsei University, Seoul, South Korea; 4 Laboratory of Host Defense, World Premier International Immunology Frontier Research Center, and Department of Host Defense, Research Institute for Microbial Diseases, Osaka University, Osaka, Japan; University of Pennsylvania, United States of America

## Abstract

Type I interferon (IFN-I) plays a critical role in the homeostasis of hematopoietic stem cells and influences neutrophil influx to the site of inflammation. IFN-I receptor knockout (*Ifnar1* ^−/−^) mice develop significant defects in the infiltration of Ly6C^hi^ monocytes in the lung after influenza infection (A/PR/8/34, H1N1). Ly6C^hi^ monocytes of wild-type (WT) mice are the main producers of MCP-1 while the alternatively generated Ly6C^int^ monocytes of *Ifnar1* ^−/−^ mice mainly produce KC for neutrophil influx. As a consequence, *Ifnar1* ^−/−^ mice recruit more neutrophils after influenza infection than do WT mice. Treatment of IFNAR1 blocking antibody on the WT bone marrow (BM) cells *in vitro* failed to differentiate into Ly6C^hi^ monocytes. By using BM chimeric mice (WT BM into *Ifnar1* ^−/−^ and vice versa), we confirmed that IFN-I signaling in hematopoietic cells is required for the generation of Ly6C^hi^ monocytes. Of note, WT BM reconstituted *Ifnar1* ^−/−^ chimeric mice with increased numbers of Ly6C^hi^ monocytes survived longer than influenza-infected *Ifnar1* ^−/−^ mice. In contrast, WT mice that received *Ifnar1* ^−/−^ BM cells with alternative Ly6C^int^ monocytes and increased numbers of neutrophils exhibited higher mortality rates than WT mice given WT BM cells. Collectively, these data suggest that IFN-I contributes to resistance of influenza infection by control of monocytes and neutrophils in the lung.

## Author Summary

Type I interferon (IFN-I) was originally reported as a molecule that interferes with influenza virus replication. Various IFN-I inducible antiviral proteins contribute to dampening virus replication and dissemination. Thus, loss of IFN-I signaling attenuates antiviral response and aggravates disease. Recent studies suggest the possible role of IFN-I in hematopoiesis, which subsequently might have an effect on the immune cell response at the site of infection. Indeed, IFN-I signaling-defective mice have been shown to develop aberrant cell populations. The aim of this current study was to clarify the mechanisms of IFN-I signaling in the regulation of monocytes and neutrophils. We show that IFN-I is directly involved in monocyte differentiation and that loss of IFN-I signaling allows mice to generate monocytes whose gene profile is significantly different. We found that monocytes are an important source of chemokines for further monocyte recruitment, but IFN-I-defective monocytes produce chemokines for neutrophil recruitment. As a result, mice lacking IFN-I signaling recruit more neutrophils and a reduced number of alternatively generated monocytes. Thus, our findings indicate that authentic monocyte differentiation, which requires IFN-I signaling, is critical in controlling neutrophils and protecting mice against influenza virus infection.

## Introduction

Type I interferons (IFN-I) are produced by different cell types including alveolar macrophages (AM), plasmacytoid dendritic cells, and epithelial cells following virus infection in the lung [1], [2]. These IFN-I cytokines engage a unique heterodimeric IFN-α receptor (IFNAR) to induce various antiviral effectors [3]. IFN-I-related antiviral effectors, including protein kinase PKR [4], 2′-5′-oligo A synthetase [5], and Mx-GTPase [6], control influenza virus infection by their own or in cooperation with various other signaling pathways [7], [8]. Even though influenza NS1 protein provides antagonistic properties against IFN-I-inducible antiviral proteins, which help virus to circumvent host barriers [9], increased susceptibility in *Ifnar1* ^−/−^ mice indicates that IFN-I signaling still plays a significant role in protecting the host after influenza infection *in vivo* [10], [11].

Monocytes emigrate from bone marrow (BM) thorough CCR2 receptor-mediated signaling and then monocyte-derived cells mediate inflammatory responses against influenza infection [12], [13]. Because of the important function of these monocytes, IFN-I signaling-mediated monocyte differentiation should be examined to better understand the regulation of leukocyte differentiation at large. In accordance, one recent study showed that IFN-I signaling triggers hematopoietic stem cell (HSC) proliferation [14]. Similarly, mice lacking IFN regulatory factor-2, a suppressor of IFN-I signaling, fail to maintain quiescent HSC [15]. These studies of the effect of IFN-I on regulation of cell homeostasis may explain different cell constitutions of *Ifnar1* ^−/−^ mice. However, specific cell populations directly affected by IFN-I during hematopoiesis and their contributions toward unique cell composition in peripheral tissues are not yet described. Thus, the role of IFN-I on the regulation of overall monocyte differentiation and infiltration into inflamed tissue needs to be analyzed in depth.

Although neutrophils are universally accepted as important in bacterial infection resistance [16], their role in viral infection remains controversial. Tate and colleagues reported that mice undergo more pronounced disease when neutrophils are absent [17], [18]. However, depletion of neutrophils not only results in increased viral burden but also in decreased lung inflammation, indicating that neutrophils contribute to control virus dissemination but may augment overall pathogenesis [19]. Clinically, excessive neutrophil recruitment, especially after highly pathogenic avian H5N1 or 1918 pandemic influenza infection, seems to play a detrimental role in acute lung injury [20], [21]. Thus, neutrophil infiltration seems to be closely related to tissue damage following infection as well as to inflammation, and the feed-back regulation of neutrophil recruitment to the site of infection needs to be tightly regulated.

In the current study, we adopted the influenza infection model and found that *Ifnar1* ^−/−^ mice undergo more acute and severe inflammation than B6 wild-type (WT) mice. IFN-I was directly involved in Ly6C^hi^ monocyte differentiation from its precursor and these Ly6C^hi^ monocytes exclusively provided MCP-1 in the lung after influenza infection. Further, *Ifnar1* ^−/−^ mice with defects in monocyte maturation produced excess KC chemokine and developed high mortality and severe neutrophilia when compared with WT mice. Our results suggest that IFN-I is required to resist influenza infection by orchestrating the leukocyte population in the lung and chemokines produced by those cells.

## Results

### *Ifnar1* ^−/−^ mice develop more acute and severe lung inflammation than WT mice

**Figure 1:**
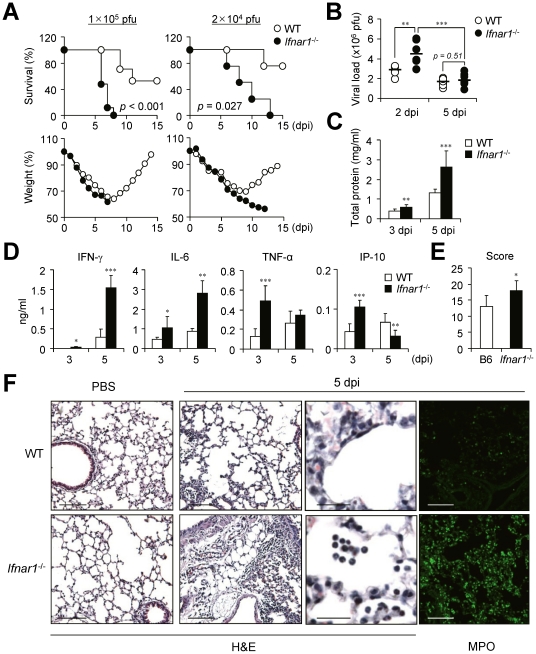
*Ifnar1* ^−/−^ mice are more susceptible to influenza infection than wild-type (WT) mice. (A) Survival and weight loss of WT and *Ifnar1* ^−/−^ mice after intranasal (i.n.) infection with 1×10^5^ pfu or 2×10^4^ pfu PR8 virus. (B) Viral load was measured by plaque assay in the lung of mice infected with 1×10^5^ pfu PR8 virus at 2 (*n* = 6) and 5 (*n* = 11) days post infection (dpi). Total protein (C) and cytokine (D) levels were determined in the BALF of PR8-infected mice at 3 and 5 dpi. (C, D) Values are mean + SD and results are representative of three independent experiments with *n*>5 for each group. Histopathological scores (E) and representative photos (F) of lungs from WT and *Ifnar1* ^−/−^ mice (3 mice/group) after infection with 1×10^5^ pfu PR8 virus and staining with H&E or MPO. Bars: 100 µm or 25 µm (enlarged pictures).

Because many previous studies indicate a crucial role for IFN-I in host defense against influenza infection, we looked for crucial regulatory factors that are mainly regulated by IFN-I after influenza infection using *Ifnar1^−/−^* mice of B6 background. First we challenged *Ifnar1* ^−/−^ and WT mice with a lethal dose (1×10^5^ pfu) of PR8 virus. The *Ifnar1^−/−^* mice started to die 5 days post infection (dpi) and all were dead within 8 dpi while approximately 50% of WT mice survived (Figure 1A). When we decreased the challenge dose of PR8 virus (2×10^4^ pfu), virus infection killed *Ifnar1* ^−/−^ mice from 5 dpi and no mice survived at 13 dpi while 80% of WT B6 mice survived (Figure 1A). WT mice started to regain body weight 8 or 9 days after infection while *Ifnar1* ^−/−^ mice continued to lose weight until they died (Figure 1A). Since the contribution of IFN-I in viral clearance is controversial [22], [23], we next addressed viral titer in the lung at 2 and 5 dpi with influenza PR8 virus (1×10^5^ pfu). Intriguingly, *Ifnar1* ^−/−^ mice showed higher viral titer in the lung than WT mice at 2 dpi but not at 5 dpi (Figure 1B). However, total protein levels were significantly higher in the bronchoalveolar lavage fluid (BALF) of *Ifnar1* ^−/−^ mice at 5 dpi than in WT mice (Figure 1C). Of note, significant levels of IL-6, TNF-α and IP-10 were determined at 3 dpi in the BALF of *Ifnar1* ^−/−^ mice and high levels IFN-γ and IL-6 at 5 dpi when compared with levels in WT mice (Figure 1D). Lung histopathology of WT and *Ifnar1* ^−/−^ mice after H&E staining revealed more edema, alveolar hemorrhage, alveolar wall thickness, and neutrophil infiltration in *Ifnar1* ^−/−^ mice than in WT mice at 5 dpi (Figure 1E and 1F). Staining specifically for myeloperoxidase (MPO), most abundantly present in the granules of neutrophils, confirmed increased numbers of MPO^+^ neutrophils in the lung of *Ifnar1* ^−/−^ mice after influenza infection (Figure 1F).

### IFN-I signaling is involved in leukocyte infiltration in the lung after influenza infection

**Figure 2:**
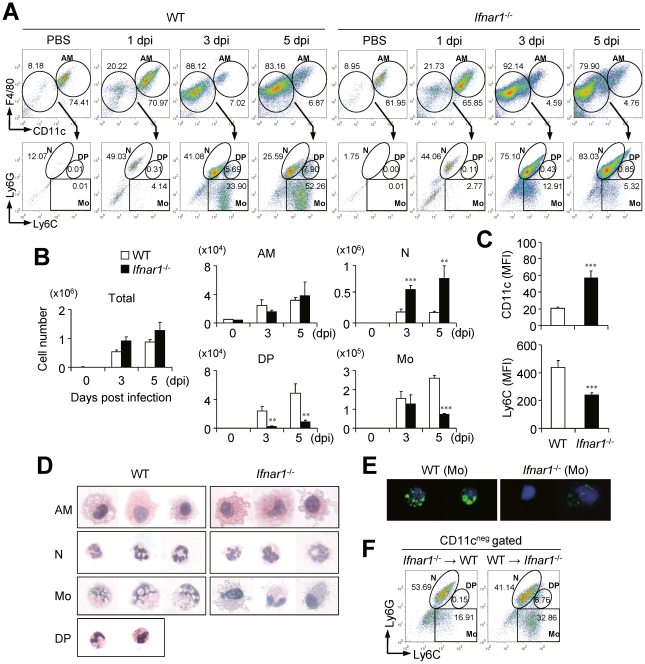
*Ifnar1* ^−/−^ mice develop different cell population profiles in the lung after influenza infection. Mice were infected i.n. with 1×10^5^ pfu PR8 virus. (A) Cells were obtained from BALF at 1, 3, and 5 days post infection (dpi) and cellular compositions were determined by flow cytometry. AM, alveolar macrophages; N, neutrophils; DP, double-positive cells; Mo, monocytes. Numbers indicate cell population percentages within gates. (B) Absolute cell numbers of each subset in the BALF of mice pre and post infection. (C) CD11c and Ly6C expression levels in the monocytes isolated from the BALF of WT and *Ifnar1* ^−/−^ mice at 5 dpi. MFI; mean fluorescence intensity. Indicated cell subsets were isolated from the lung of WT and *Ifnar1* ^−/−^ mice at 5 dpi and stained with H&E (D) or Nile red (E) after cytospin. Original magnification ×40. (F) Recipient WT and *Ifnar1* ^−/−^ mice were lethally irradiated and reconstituted with bone marrow (BM) cells from *Ifnar1* ^−/−^ and WT mice, respectively. Cell populations in the BALF isolated from recipient WT and *Ifnar1* ^−/−^ mice at 3 dpi were analyzed. Values are mean + SD and results are representative of three independent experiments with *n*>5 for each group.

As *Ifnar1* ^−/−^ mice of B6 background exhibited severe pathology in terms of hyper secretion of pro-inflammatory cytokines and neutrophilia in lung after infection with influenza virus, we examined profiles of infiltrated cell populations in BALF in a time-dependent manner. From 1 dpi with influenza virus, predominant numbers of neutrophils (Ly6C^int^Ly6G^+^) were infiltrated into the lung of WT and *Ifnar1* ^−/−^ mice (Figure 2A). Of note, the proportion (Figure 2A) and absolute numbers (Figure 2B) of infiltrated neutrophils in BALF were much higher in *Ifnar1* ^−/−^ mice than in WT mice. Furthermore, the numbers of neutrophils in WT mice peaked at 3 dpi and decreased at 5 dpi while neutrophils in *Ifnar1* ^−/−^ mice increased until 5 dpi, when mice began to die (Figure 2B). Meanwhile, monocytes (Ly6C^+^Ly6G^−^) were gradually infiltrated into the lung of WT mice in a time-dependent manner after influenza infection (Figure 2A and 2B). However, fewer monocytes were recruited into the lung of *Ifnar1* ^−/−^ mice than in WT mice (Figure 2A and 2B) and they were Ly6C^int^ rather than Ly6C^hi^ (Figure 2C). Since Ly6C^hi^ monocytes have similar phenotype to myeloid-derived suppressor cells, which expand during cancer, inflammation, and infection [24], we tested their ability to suppress CD4^+^ T cells. However, we did not find any evidence of CD4^+^ T cell suppression by Ly6C^hi^ monocytes (Figure S1A). We next examined expression levels of co-stimulatory molecules, such as CD40, CD80, CD86, and MHCII, on surfaces of the respective cell populations, but overall these markers were not significantly different between cells isolated from WT and *Ifnar1* ^−/−^ mice (Figure S1B). We further analyzed surface expression of various markers on monocytes of infected WT and *Ifnar1* ^−/−^ mice (Figure S1C). Monocytes in the lung of infected WT and *Ifnar1* ^−/−^ mice expressed macrophage-related markers and they were negative for markers specific for dendritic cells, lymphocytes, and natural killer cells [25].

Because MyD88 signaling cooperates with IFN-I in Ly6C^hi^ monocyte recruitment in a *Listeria monocytogenes* infection model [26], we tested whether Toll-like receptor (TLR) or RIG-I-like receptor (RLR) signaling is involved in Ly6C^hi^ monocyte regulation in our model. However, mice have defects in TLR (*Myd88* ^−/−^ *Trif* ^−/−^) or RLR (*Ips-1* ^−/−^) signaling normally generated Ly6C^hi^ monocytes (Figure S2A). Although IPS-1 is involved in IFN-I expression, deletion of IPS-1 can be compensated by MyD88 signaling after influenza infection [27]. Indeed, *Ips-1* ^−/−^ mice produced IFN-α comparable to *Ips-1* ^+/+^ mice (Figure S2B). Overall, direct engagement of IFN-I signaling through IFNAR but not TLR or RLR signaling seems to play a crucial role in Ly6C^hi^ monocyte infiltration into the lung for host defense after influenza infection.

### Ly6C^pos^ monocytes from influenza-infected lung of WT and *Ifnar1* ^−/−^ mice have different characteristic features

To assess the innate immune cells of WT and *Ifnar*1^−/−^ mice in more detail, we next examined their morphologies (Figure 2D). Both AM and neutrophils in the lung seemed identical in WT and *Ifnar*1^−/−^ mice except that some neutrophils from *Ifnar*1^−/−^ mice exhibited larger size and had a more diffused nucleus, but monocytes were clearly different in the lung of WT and *Ifnar*1^−/−^ mice after influenza infection. Interestingly, Ly6C^hi^ monocytes morphologically resemble the foamy macrophages previously found in the lung of *Mycobacterium bovis* bacillus Calmette-Guérin-infected mice [28]. To confirm this, we stained Ly6C^pos^ monocytes with Nile red, which mainly stains lipid body, and found that only Ly6C^pos^ monocytes isolated from the lung of WT mice were positively stained (Figure 2E). Next we generated BM chimeric mice (WT BM into *Ifnar1* ^−/−^ mice and vice versa) to confirm whether the defect in IFN-I signaling in the hematopoietic cell lineage can trigger the alteration of monocyte phenotypes in the lung of influenza-infected mice. As a result, Ly6C^hi^ monocytes were generated in *Ifnar1* ^−/−^ recipient mice reconstituted with WT BM cells but were not detected in WT recipient mice that received *Ifnar1* ^−/−^ BM cells (Figure 2F). These suggest that IFN-I signaling in the hematopoietic cell lineage plays an indispensable role for differentiation of Ly6C^hi^ monocytes against influenza infection.

### Monocytes from WT mice produce MCP-1 while those from *Ifnar1* ^−/−^ mice produce KC against influenza infection

**Figure 3:**
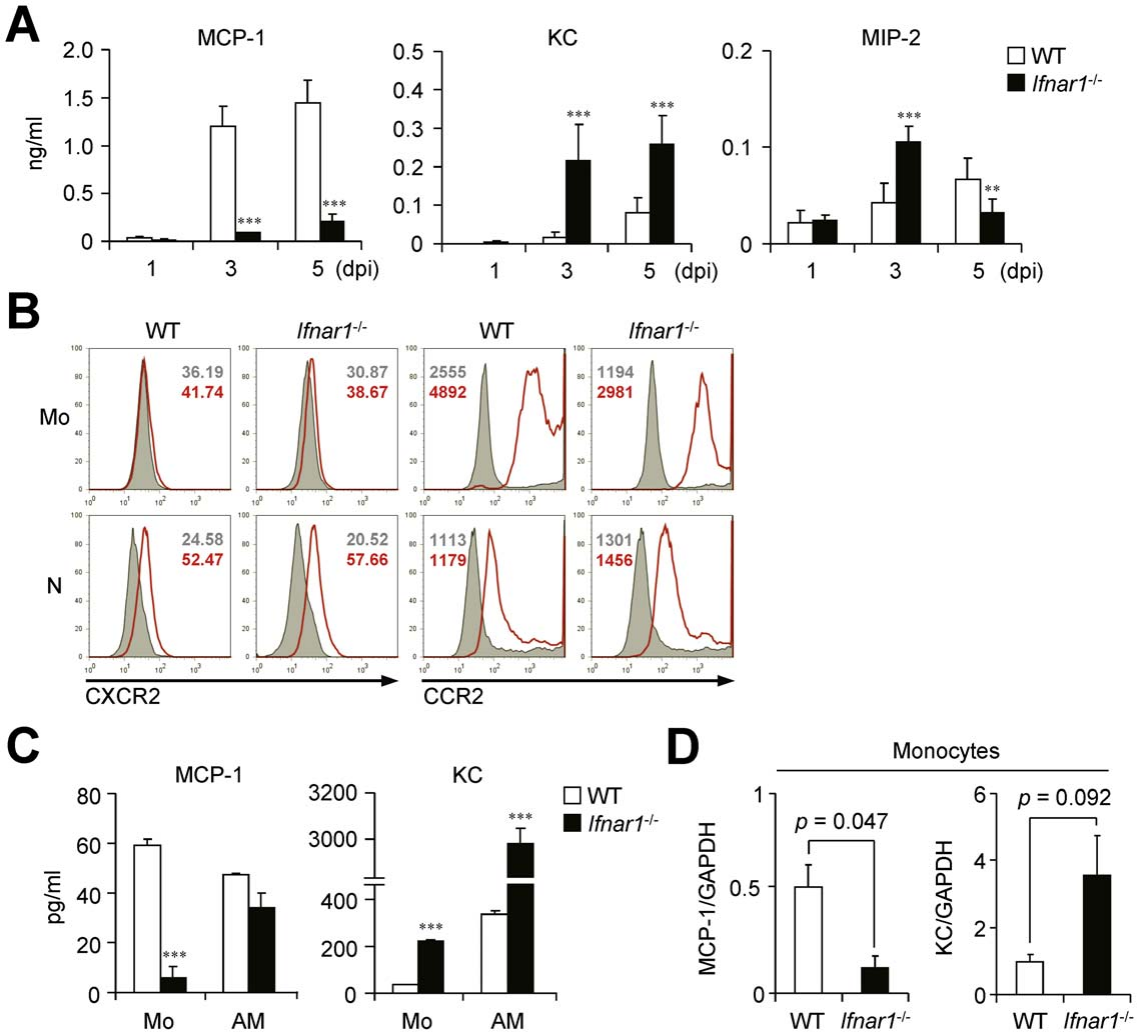
*Ifnar1* ^−/−^ mice produce significantly lower MCP-1 and higher KC than WT mice. (A) Levels of MCP-1, KC, and MIP-2 were determined in the BALF of PR8 virus (1×10^5^ pfu)-infected WT and *Ifnar1* ^−/−^ mice at 1, 3, and 5 dpi. Values are mean + SD and results are representative of three independent experiments with *n*>4 for each group. (B) Expression patterns of chemokine receptors, CXCR2 and CCR2, on monocytes (Mo) and neutrophils (N) were analyzed by flow cytometry. Histograms are representative of two independent experiments and numbers indicate MFI. (C) Monocytes (Mo) and alveolar macrophages (AM) were isolated using FACS Aria and plated onto 96-well plates. After culture for 4 h, MCP-1 and KC levels in the supernatants were analyzed. Data are representative of three independent experiments and values are mean + SD of three independently cultured wells. (D) mRNA expression levels of MCP-1 and KC were analyzed by gene chip data using monocytes from WT and *Ifnar1* ^−/−^ mice 5 dpi. The expression levels were normalized by GAPDH expression levels. Data are mean values of two independent experiments.

We next measured the chemokines responsible for leukocyte migration against influenza infection in the BALF at different time points. Of note, expression levels of MCP-1 and KC, the main chemokines for CCR2- and CXCR2-dependent cell recruitment, respectively [29], [30], were very different in the BALF of WT and *Ifnar1* ^−/−^ mice (Figure 3A). The *Ifnar1* ^−/−^ mice had significantly lower levels of MCP-1 than the WT mice but had predominant levels of KC at 3 and 5 dpi (Figure 3A). In addition, MIP-2, another well-known molecule for neutrophil recruitment [31], was significantly higher in *Ifnar1* ^−/−^ mice at 3 dpi (Figure 3A). Both Ly6C^hi^ and Ly6C^int^ monocytes obtained from the lung of WT and *Ifnar1* ^−/−^ mice expressed CCR2 but not CXCR2 (Figure 3B), suggesting the critical role of MCP-1 for monocyte recruitment into influenza-infected lung.

To directly compare chemokine expression by individual cell populations, we sorted Ly6C^pos^ monocytes from influenza-infected lung of WT and *Ifnar1* ^−/−^ mice and then cultured them *in vitro*. Although both monocytes and AMs isolated from WT mice produced MCP-1 (Figure 3C), recovered Ly6C^hi^ monocytes were quantitatively overwhelming over AMs (2.6×10^5^ vs. 3.1×10^4^ in BALF per mouse at 5 dpi) (Figure 2B). These findings suggest that Ly6C^hi^ monocytes are the main producer of MCP-1 among leukocytes in WT mice after influenza infection. In contrast, in *Ifnar1* ^−/−^ mice, KC was highly produced by both Ly6C^int^ monocytes and AMs, but Ly6C^int^ monocytes secreted significantly less MCP-1 than WT Ly6C^hi^ monocytes (Figure 3C). It is noteworthy that types of chemokines expressed in monocytes from WT and *Ifnar1* ^−/−^ mice were in complete contrast; gene expression profiles analyzed by gene chip experiments support these results (Figure 3D). Collectively, these data suggest that IFN-I is crucial for determining monocyte characteristics that dramatically influence chemokine production.

### Monocytes from WT and *Ifnar1* ^−/−^ mice show different gene profiles

**Figure 4:**
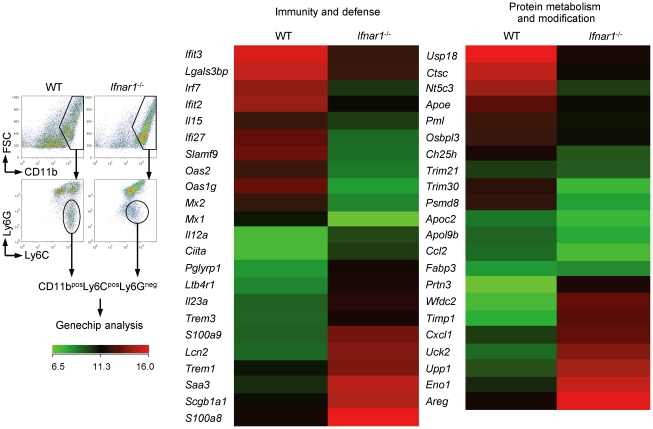
Monocytes of *Ifnar1* ^−/−^ mice express different patterns of genes. WT and *Ifnar1* ^−/−^ mice were infected i.n. with 1×10^5^ pfu PR8 and monocytes were sorted. Total RNA was extracted and gene expression profiles were analyzed on gene chip. Data are representative of two independent experiments.

For macroscopic comparison between monocytes recruited in the lung of influenza-infected WT and *Ifnar1* ^−/−^ mice, we performed gene chip analysis (Figure 4). As expected, IFN-I-regulated genes (e.g., *Mx*, *Oas*, *Irf*, etc.) were significantly decreased in monocytes isolated from *Ifnar1* ^−/−^ mice. Of the genes elevated in Ly6C^int^ monocytes isolated from *Ifnar1* ^−/−^ mice, *S100a8*/*S100a9* and *Trem1* are associated with inflammatory responses in various diseases [32], [33]. In contrast, negative regulators of inflammation, *Trim21* and *Trim30* [34], [35], were up-regulated in Ly6C^hi^ monocytes isolated from WT mice when compared to Ly6C^int^ monocytes from *Ifnar1* ^−/−^ mice. These inflammation-biased gene expressions by Ly6C^int^ monocytes may explain the higher susceptibility of *Ifnar1* ^−/−^ mice to influenza infection. Ly6C^hi^ monocytes from WT mice were also superior in expressing genes involved in lipid metabolism (e.g., *Apoe*, *Apoc2*, etc.). Interestingly, the 1918 pandemic influenza virus was found to block lipid metabolism as part of its evasion strategy against antiviral responses [36]. Furthermore, influenza infection causes prominent inflammation in *Apoe* ^−/−^ mice [37], indicating that a defect in lipid metabolism in *Ifnar1* ^−/−^ mice might contribute to worsen inflammation. Collectively, we confirmed that monocytes from WT and *Ifnar1* ^−/−^ mice have significantly different characteristics and that lack of IFN-I signaling changes gene expression bias to augment inflammation.

### IFN-I signaling is required for Ly6C^hi^ monocyte generation

**Figure 5:**
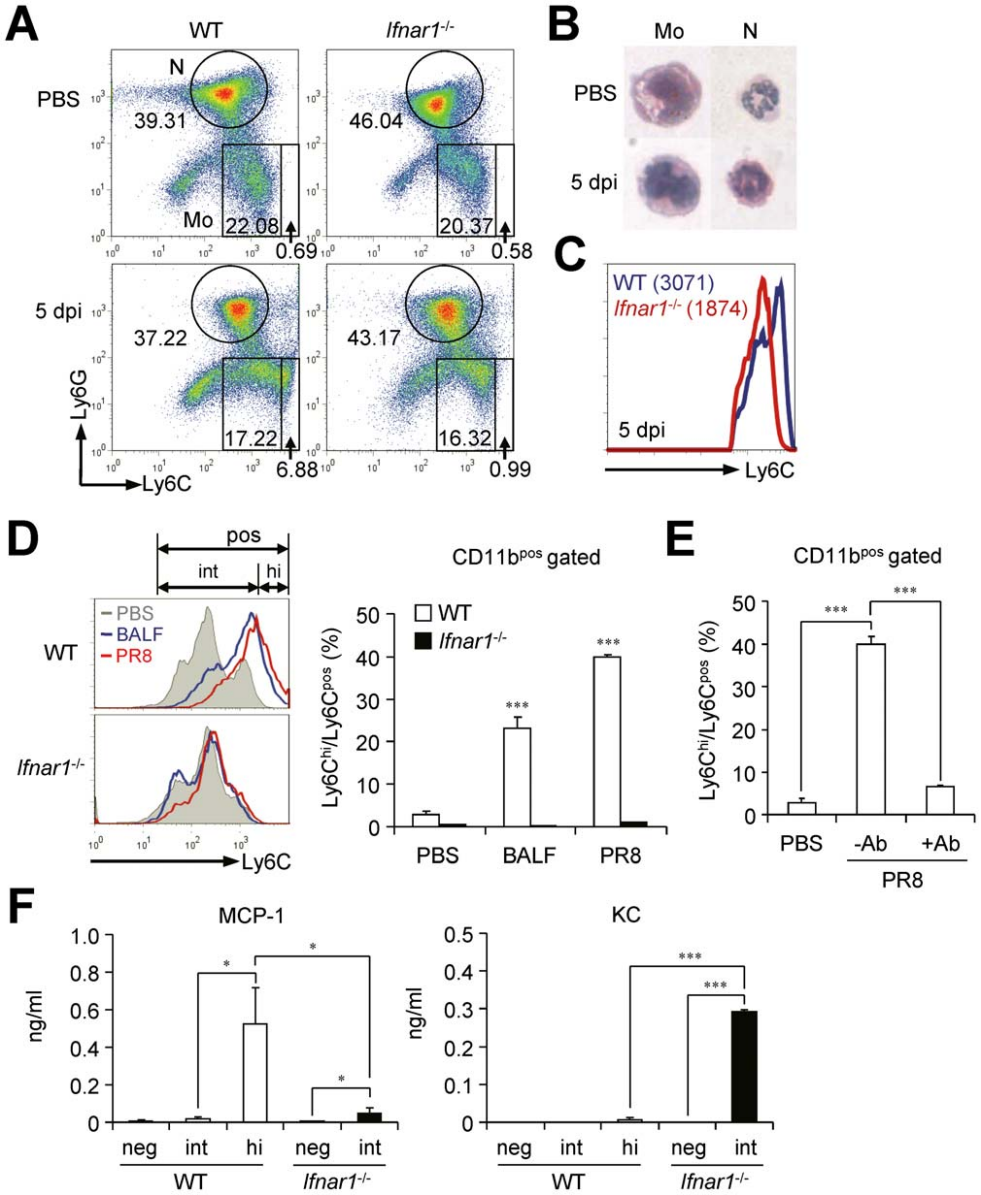
*Ifnar1* ^−/−^ bone marrow (BM) cells are unable to differentiate into MCP-1-producing Ly6C^hi^ monocytes. (A) BM cells were isolated from WT and *Ifnar1* ^−/−^ mice pre- and post-influenza infection and analyzed by flow cytometry. (B) Monocytes (Mo) and neutrophils (N) were isolated, cytospun, and stained by H&E (×40). (C) MFI of Ly6C expression of monocytes in the BM of WT and *Ifnar1* ^−/−^ mice following influenza infection (1×10^5^ pfu). Mean values are in parenthesis. (D) BM cells from naïve WT and *Ifnar1* ^−/−^ mice were prepared and stimulated *in vitro* with BALF from PR8 virus (1×10^5^ pfu)-infected WT mice or with PR8 virus (0.5 multiplicity of infection) alone. After 5 days of culture, subsets of Ly6C^hi^ and Ly6C^int^ were counted and the percentage of Ly6C^hi^ among Ly6C^pos^ was determined. (E) After BM cells were prepared from naïve WT mice, cells were infected with PR8 virus and cultured for 5 days with/without anti-IFNAR1 blocking Ab (100 ng/ml). (F) BM cells infected with PR8 and cultured *in vitro* for 5 days. Cultured cells were further sorted into Ly6C^neg^, Ly6C^int^, and Ly6C^hi^. Each cell subset was cultured *in vitro* for 4 h and chemokine levels in the supernatant were analyzed. Values are mean + SD and data are representative of at least two independent experiments.

Since the different gene expression patterns in Ly6C^pos^ monocytes of WT and *Ifnar1* ^−/−^ mice after influenza infection can be ascribable to altered monocyte differentiation from their precursors, we next analyzed BM where hematopoiesis occurs and provides common monocyte precursors. The proportion of Ly6C^hi^ monocytes was significantly lower (6.9±1.9 vs. 1.0±0.3%) in the BM of *Ifnar1* ^−/−^ mice than in WT mice, while the proportion of Ly6C^int^ monocytes was comparable to WT mice (17.2±0.6 vs. 16.3±1.5%) at 5 dpi (Figure 5A). However, there were no significant differences in cell morphology between WT and *Ifnar1* ^−/−^ mice (data not shown), and Ly6C^pos^ monocytes of WT mice did not show lipid bodies unlike Ly6C^hi^ monocytes in the lung post influenza infection (Figure 5B). Because we found higher levels of Ly6C expression in BM monocytes from WT than in *Ifnar1* ^−/−^ mice at 5 dpi (Figure 5C), we further assessed whether IFN-I signaling can directly affect differentiation of naïve BM cells *in vitro*. When BM cells were stimulated with WT BALF collected at 5 dpi or directly infected with influenza virus, WT BM cells were able to differentiate into Ly6C^hi^ monocytes but *Ifnar1* ^−/−^ BM could not (Figure 5D). To confirm that this maturation defect of Ly6C^hi^ monocytes from *Ifnar1* ^−/−^ BM is due to lack of IFN-I signaling, we co-cultured PR8-infected WT BM cells with or without anti-IFNAR1 blocking antibody. When treated with anti-IFNAR1 antibody, WT BM cells failed to differentiate into Ly6C^hi^ monocytes (Figure 5E). Importantly, these Ly6C^hi^ monocytes derived from WT BM stimulated by influenza virus dominantly produced MCP-1 when compared to Ly6C^int^ monocytes derived from WT or *Ifnar1* ^−/−^ BM, while *Ifnar1* ^−/−^ Ly6C^int^ monocytes produced robust KC instead (Figure 5F). From this observation, we suggest that absence of Ly6C^hi^ cells in lung of *Ifnar1* ^−/−^ mice results from failure in differentiation of their precursors due to lack of IFN-I signaling.

### IFN-I signaling on hematopoietic cells is required to resist influenza infection

**Figure 6:**
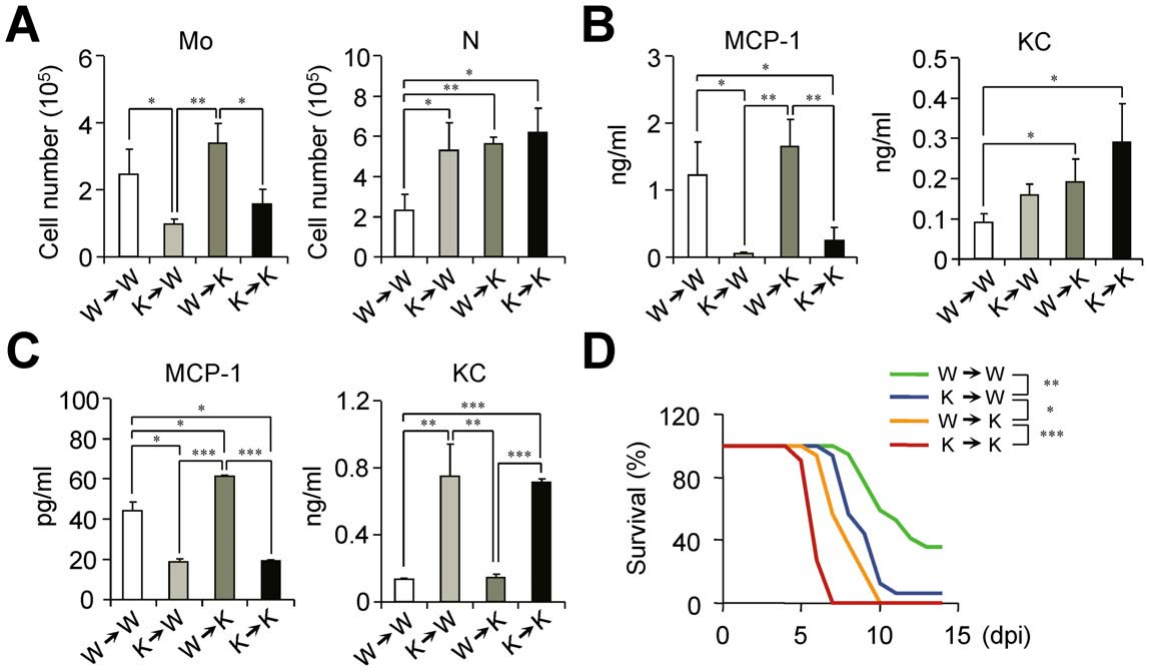
IFN-I signaling on hematopoietic cell lineage helps protect mice from lethal influenza infection. Bone marrow (BM) cells from naïve WT (W) or *Ifnar1* ^−/−^ (K) mice were grafted to lethally irradiated WT or *Ifnar1* ^−/−^ recipient mice. (A–C) Sets of BM grafted mice were challenged with influenza PR8 virus (1×10^5^ pfu) and analyzed at 3 dpi. (A) Number of monocytes (Mo) and neutrophils (N) were determined. (B) Expression levels of MCP-1 and KC were measured in BALF. (C) Monocytes in the lung were pooled and cultured for 4 h to measure chemokine production *in vitro*. Values are mean + SD of triplicate experiments with *n*>4 for each group. (D) Mice were challenged with lethal dose of influenza PR8 (1×10^5^ pfu) and survival was monitored for 2 weeks. W→W (*n* = 17), K→W (*n* = 16), W→K (*n* = 16), and K→K (*n* = 11). Data were pooled from three independent experiments.

To clarify the origin and roles of monocytes in depth in the absence of IFN-I signaling, we used BM chimeric mice. *Ifnar1* ^−/−^ recipient mice reconstituted with WT BM cells (W→K) have significantly more Ly6C^hi^ monocytes than K→K (*Ifnar1* ^−/−^ donor and *Ifnar1* ^−/−^ recipient) mice (Figure 6A). Concomitantly, the MCP-1 level in the BALF was correlated with Ly6C^hi^ monocytes (i.e., W→W and W→K) after influenza infection (Figure 6B). The fact that K→W mice elicited no higher level of MCP-1 than found in the BALF of K→K mice after influenza infection suggests that radio-resistant WT parenchymal cells do not play a major role in MCP-1 production (Figure 6B). In addition, Ly6C^hi^ monocytes derived from W→W and W→K mice produce MCP-1 efficiently following *in vitro* culture (Figure 6C), clearly indicating that Ly6C^hi^ monocytes derived from WT BM mice exclusively produce MCP-1 in response to influenza infection. To the contrary, although we saw a distinct correlation between KC production with IFN-I deficiency in monocytes (Figure 6C), W→K chimera mice still had significantly augmented KC in BALF even with high levels of MCP-1 and Ly6C^hi^ monocytes (Figure 6B). Thus it seems likely that the complete regulation of KC also needs the IFN-I-dependent signaling pathway in cells other than monocytes or there may be another regulator for KC in the absence of IFN-I signaling.

Finally, we sought to find the relationships between the defect in Ly6C^hi^ monocyte generation and the susceptibility of chimeric mice against influenza infection. The loss of intrinsic Ly6C^hi^ monocytes in K→W mice resulted in the increased susceptibility of mice against influenza infection as compared with W→W mice (Figure 6D). In addition, the W→K chimera mice showed less susceptibility to influenza infection than did K→K mice with the restoration of Ly6C^hi^ monocytes (Figure 6D). To see whether neutrophils can augment inflammation and tissue damage, we isolated neutrophils from infected *Ifnar1* ^−/−^ mice and transferred them to naïve WT mice (Figure S3A). Then effect of neutrophil transfer on the lung inflammation was observed without additional infection or any treatment. Histologically, recipient WT mice had destruction in epithelial layers and showed inflammatory lesions while PBS-treated mice did not at 2 days after transfer (Figure S3B). Inflammatory cytokines in the BALF were also induced after neutrophil transfer, indicating activated neutrophil itself can cause inflammation (Figure S3C). Thus, we suggest that excess neutrophils can worsen disease even if there are good reasons for recruitment after virus infection. Even though parenchymal cells appear to maintain host defense, our data clearly show the importance of IFN-I signaling in hematopoietic cells in protection against influenza infection.

## Discussion

**Figure 7:**
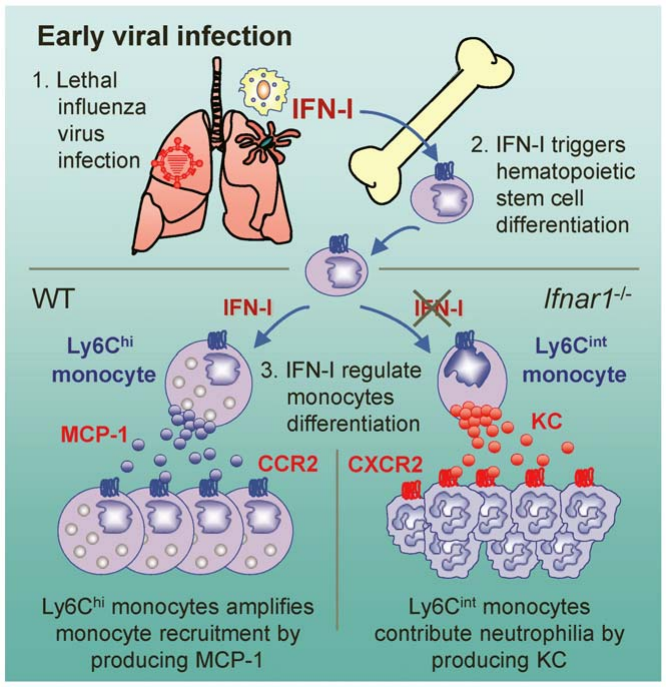
Suggested model for the role of IFN-I on monocyte and neutrophil regulation. After influenza infection, hematopoietic stem cell differentiation might be triggered by IFN-I and then MCP-1-producing Ly6C^hi^ monocytes are recruited to the lung. In the absence of IFN-I signaling, Ly6C^int^ monocytes are alternatively generated and produce KC, which might in turn aggravates lung pathology by attracting excess neutrophils to lung.

Despite numerous previous studies focused on the direct antiviral nature of IFN-I, the data we present here suggest that IFN-I-dependent generation of Ly6C^hi^ monocytes after influenza infection might be critical for the attenuation of neutrophil infiltration and hence prevent severe tissue damage caused by uncontrolled inflammation (Figure 7). Ly6C^hi^ monocytes in the lung were previously reported as TNF/iNOS-producing dendritic cells [38] or inflammatory monocytes [12], [39]. In our observations, however, Ly6C^hi^ monocytes were closest in morphology to the highly vacuolated foamy macrophages, which were found in granulomas in the lung of *Mycobacterium bovis*-infected mice [28].

Previously, it was proposed that there is no up-regulation of MCP-1 in the absence of IFN-I signaling; as a consequence, without up-regulation there will be fewer Ly6C^hi^ monocytes in various tissues [26], [40], [41]. We show here that only mice reconstituted with WT BM cells (W→W and W→K), but not with *Ifnar1* ^−/−^ BM cells (K→K and K→W), generated Ly6C^hi^ monocytes and produced high amounts of MCP-1 in the BALF against influenza infection. Further, BM cells from *Ifnar1* ^−/−^ mice were unable to differentiate into MCP-1-producing Ly6C^hi^ monocytes by *in vitro* stimulation, indicating that IFN-I signaling on hematopoietic cells is required for differentiating Ly6C^hi^ monocytes. Our findings are the first to delineate a notion that MCP-1 is mainly produced by Ly6C^hi^ monocytes, which are regulated by IFN-I against influenza infection. Additionally, it seems plausible that AMs and other cells (e.g., pulmonary epithelial cells) contribute to produce MCP-1 for initial monocyte recruitment before Ly6C^hi^ monocytes are heavily recruited after virus infection.

MCP-1 is a chemokine that has great importance in CCR2-mediated monocyte recruitment after lung inflammation [42]. Although other chemokines such as MCP-2, MCP-3, and MCP-4 also contribute to attract CCR2-expressing monocytes after influenza infection, overexpression of MCP-1 results in elevated monocyte recruitment [12]. In this regard, MCP-1-deficient mice show significantly reduced macrophage infiltration [43]. Moreover, MCP-1 is capable of activating AM [44] and of blocking MCP-1-augmented damage on epithelial cells after influenza infection [45]. Thus, Ly6C^hi^ monocytes might play an important role for host defense against influenza infection by mediating MCP-1.

Unlike MCP-1 whose level was dramatically decreased in *Ifnar1* ^−/−^ mice, KC was significantly increased after influenza infection. We observed that W→K and K→W chimeric mice produced intermediate KC when compared to W→W and K→K mice, suggesting that KC can be produced by both radio-sensitive and -resistant cells. AMs, which produced KC *in vitro*, seemed especially important producers as did radio-resistant cells (e.g., epithelial cells) as proposed by others [46]. Since KC with MCP-1 is reversely correlated in BALF and culture supernatant with monocytes from the lung of WT and *Ifnar1* ^−/−^ mice, further studies are needed to elucidate a putative role of IFN-I-dependent Ly6C^hi^ monocytes on neutrophil regulation as well as possible feedback regulation of KC by MCP-1 production.

Previously it was proposed that Ly6C^int^ monocytes were converted from Ly6C^hi^ monocytes after migration to the site of inflammation [41]. Those activated forms of Ly6C^int^ monocytes were characterized by high CX_3_CR1 but no CCR2 expression [47]. In our study, however, Ly6C^int^ monocytes generated in influenza-infected *Ifnar1* ^−/−^ mice expressed high levels of CCR2. Thus, it seems likely that Ly6C^int^ monocytes in *Ifnar1* ^−/−^ mice could be an alternative to Ly6C^hi^ monocytes generated under IFN-I-deficient conditions rather than a different cell subset of Ly6C expressing monocytes.

It has been reported that the spleen also stores monocytes and these cells deploy to inflammatory sites to regulate inflammation [25]. To address this issue, we compared characteristics of monocytes in the spleen before and after influenza infection (Figure S4A). Interestingly, Ly6C expression level in the monocytes was much lower in the spleen of *Ifnar1* ^−/−^ mice than in WT mice in the steady-state condition (Figure S4B). The number of Ly6C^hi^ monocytes stored in the spleen was also decreased in *Ifnar1* ^−/−^ mice compared to WT mice, whereas the number of neutrophils was comparable before influenza infection (Figure S4C). We speculate that the reduced numbers of WT and *Ifnar1* ^−/−^ monocytes after influenza infection in the spleen may indicate the spleen is a reservoir that provides monocytes to the lung.

After finding that IFN-I signaling is involved in Ly6C^hi^ generation, we next sought to clarify the relationships between the defect in Ly6C^hi^ monocytes and susceptibility to influenza infection. Viral titer in lethally infected lung culminated around 2 dpi and continuously decreased. Although *Ifnar1* ^−/−^ mice were intact in viral clearance and showed similar viral burden at 5 dpi compared to WT mice, they developed severe inflammation and consequently higher susceptibility. In previous studies, attenuated inflammation provided resistance to influenza infection even with increased viral burden [12], [48]. These findings indicate that virus-induced inflammation could be more critical than viral burden itself in the course of influenza pathology. Thus, we suggest that severe and acute lung inflammation in *Ifnar1* ^−/−^ mice, especially with uncontrolled accumulation of neutrophils due to massive KC production, contributes to increased susceptibility of those mice to influenza infection.

Accumulation of neutrophils is one of the most important events during acute respiratory distress syndrome [49]. Neutrophils are generally thought to aggravate lung injury after influenza infection [50], especially in severe infections such as those we assessed in our study. To address the contribution of neutrophils to virus-induced airway hyperresponsiveness as proposed by others [51], [52], we used CXCR2 blocking Ab and CXCR2 antagonist SB225002 [29], [53]. These materials partially dampened neutrophil responses when moderate or low doses of influenza were given, but they could not efficiently block massive influxes of neutrophils after lethal influenza infection in *Ifnar1* ^−/−^ mice (data not shown). However, we found that neutrophils can augment inflammation and tissue damage (Figure S3); also loss of Ly6C^hi^ monocytes in K→W mice augmented neutrophil infiltration compared to W→W mice, indicating the balance between neutrophils and Ly6C^hi^ monocytes are reversely correlated. Moreover, when chimeric mice were lethally challenged, susceptibility of these mice was directly proportional to neutrophil numbers. Our data suggest that uncontrolled neutrophils may aggravate the outcome of excess inflammation against virus infection. Even though neutrophils are thought to augment inflammation and make disease worse, we still must consider their protective role. We showed *Ifnar1* ^−/−^ mice had higher peak virus titer but were able to successfully control virus replication. Regarding previous report that neutrophils can limit virus replication [19], it seems plausible that excessively recruited neutrophils may contribute to observed virus elimination in *Ifnar1* ^−/−^ mice. However, several lines of evidence lead us to speculate that destructive trait of neutrophils dominated over their positive role during severe and acute viral pneumonia.

IFN-I is used to treat several diseases, including hepatitis B virus infection [54], chronic hepatitis C virus infection [55], and multiple sclerosis [56]. Since IFN-I, which is produced by virus infection, can migrate and affect BM to switch on the production of functional monocytes [57], therapeutically administrated IFN-I can communicate with BM leukocytes. This possibility suggests that clinical uses of IFN-I should be investigated in terms of modified patient leukocyte profiles, especially in those receiving prolonged IFN-I therapy.

Influenza virus NS1 protein antagonizes IFN-I responses, and influenza virus lacking the NS1 gene replicates inefficiently in tissue culture and normal egg culture conditions and shows attenuated phenotype in WT mice; however, it replicates far more efficiently in IFN-deficient Vero cells and pathogenic in *Stat1* ^−/−^ mice [58]. In the clinical context, it is important to note that the NS1 protein of highly pathogenic viruses, such as H5N1 avian influenza and the 1918 pandemic influenza virus, has stronger suppressive effects on IFN-I [59], [60]. These viruses also are involved in more acute recruitment of neutrophils, severe lung injury, and aggressive inflammatory cytokine production (so-called cytokine storm) as found in *Ifnar1* ^−/−^ mice [20], [61], [62]. Thus our results in *Ifnar1* ^−/−^ mice merit further study to help understand the pathogenesis of highly pathogenic influenza virus.

Our findings show the specific function of IFN-I on Ly6C^hi^ monocyte differentiation and address the impact of this event in the lung after influenza infection. Further studies on regulation of neutrophils and Ly6C^hi^ monocytes by potential pandemic virus may provide insight that will prove useful for development of novel therapeutic targets.

## Materials and Methods

### Ethics statement

All animal experiments were approved by the Institutional Animal Care and Use Committee of the International Vaccine Institute (Approval No: PN 1003), and all experiments were carried out in strict accordance with the Guide for the Care and Use of Laboratory Animals, Institute of Laboratory Animal Resources Commission on Life Sciences National Research Council, USA. All experiments were performed under anesthesia with a mixture of ketamine (100 mg/kg) and xylazine (20 mg/kg), and all efforts were made to minimize suffering.

### Mice and virus infection

C57BL/6 (B6) mice were purchased from Charles River Laboratories (Orient Bio Inc., Sungnam, Korea). *Ifnar1* ^−/−^ mice (B6 background) were purchased from B&K Universal Ltd. (Hull, U.K.). To generate chimeric mice, naïve B6 and *Ifnar1* ^−/−^ recipient mice were lethally irradiated with 960 rad and donor BM cells (1×10^7^) were reconstituted by intraperitoneal injection. Chimeric mice were maintained for at least 8 weeks and chimerism was assessed by IFNAR1 expression on Gr-1^+^ cells in serum. Mice were infected intranasally (20 µl) with influenza A/PR/8/34 (PR8, H1N1) virus after anesthesia.

### Sample preparation

To obtain BALF, tracheas were cannulated after exsanguination and lungs were washed with 1 ml of PBS. BALF samples were centrifuged (800×g, 5 min) to isolate cells and supernatants were centrifuged again (13,000×g, 1 min) to completely remove remaining cells. BM cells obtained from femurs and tibias and red blood cells were removed before analysis. In some experiments, cells were cultured *in vitro* (1×10^5^ cells/well) for 4 h in RPMI (Gibco, Auckland, New Zealand) supplemented with 10% FBS (Gibco) to measure chemokines. Cultured cells were removed from supernatants by centrifugation (2,300×g, 3 min) and supernatants were used for further analysis.

### Virus plaque titration

Total lung was removed and homogenized to prepare lung extracts in 1 ml of PBS (pH 7.4). Confluent Madin-Darby canine kidney (MDCK) cells were washed with MEM (Gibco) once and treated with virus for 30 min at room temperature. After a wash with MEM, the plate was overlaid with MEM containing 1% low-melting-point agarose and 10 µg/ml of trypsin and incubated at 37°C for 3 days.

### Measurement of total protein in BALF

We measured total protein in BALF samples by BCA Protein Assay Kit (Pierce, Rockford, IL) according to the manufacturer's instructions.

### Cytokine and chemokine detection

The levels of MCP-1, IL-6, TNF-α, and IFN-γ were measured by Mouse Inflammatory Cytometric Bead Array Kit (BD Biosciences, San Jose, CA). The levels of KC, MIP-2, and IP-10 were measured by DuoSet Mouse ELISA Kit (R&D Systems, Minneapolis, MN) according to the manufacturer's instructions.

### Histology

Lungs were removed from naïve or infected B6 and *Ifnar1* ^−/−^ mice and washed using PBS before being fixed with 4% formaldehyde for 1 h at 4°C. The tissues were embedded in paraffin and stained with H&E. To detect MPO expression, tissues were dehydrated in sucrose solutions (10, 20, and 30%) after fixation and embedded in OCT compound (Sakura Finetec, Tokyo, Japan). Cryo sections (5 µm) were fixed in ice-cold acetone and blocked with FcRII/III mAb (2.4G2; BD Pharmingen, San Jose, CA) in PBS. Then, tissues were stained with FITC-conjugated anti-MPO (2D4; Abcam, Cambridge, MA) for confocal microscopy. Histopathological score was assessed by a pathologist using a blind test. As previously described [63], we used a scoring system of 20 points to evaluate the level of lung tissue destruction, epithelial cell layer damage, polymorphonuclear cell infiltration into the site, and alveolitis.

### Flow cytometry

Cells were collected from lung or BM and stained with the following antibodies: CD11c (HL3), CD11b (M1/70), Ly6C (AL-21), Ly6G (1A8), all purchased from BD Pharmingen; F4/80 (BM8) from eBioscience (San Diego, CA); and CXCR2 (242216) from R&D Systems; CCR2 (MC-21) was obtained from Prof. Matthias Mack (University of Regensburg, Germany). The cells were read by FACSCalibur (BD Biosciences) and data were analyzed by FlowJo 7.2.5 (Tree Star, Ashland, OR). In some experiments, cells were sorted using FACSAria (BD Biosciences). Cell populations in the lung were classified using these surface markers: AM (CD11c^hi^F4/80^+^), DP (Ly6C^+^Ly6G^+^), neutrophils (Ly6C^int^Ly6G^+^), Ly6C^hi^ monocytes (Ly6C^hi^Ly6G^−^), and Ly6C^int^ monocytes (Ly6C^int^Ly6G^−^). For analysis of BM Ly6C/Ly6G-positive cells, CD11b^+^ cells gated out and further divided depending on their Ly6C and Ly6G expressions.

### Cytospin and Nile red staining

To cytospin cells on Cytoslide (Thermo Scientific, Asheville, NC), sorted cells were centrifuged at 1,000 rpm for 10 min using CytoSpin 4 Cytocentrifuge (Thermo Scientific). Then cells were fixed and stained with H&E. For Nile red staining, stock solution (Sigma, St. Louis, MO; 0.1 mg/ml in acetone) was diluted 1∶5,000 in PBS and cells were stained for 30 min at 37°C. Samples were washed twice with Ca^2+^/Mg^2+^-free HBSS and cytospun. Then fixed specimens (3.7% formaldehyde) were stained with DAPI and washed twice before mounting.

### BM cell *in vitro* stimulation

Cells were prepared from BM of naïve WT and *Ifnar1* ^−/−^ mice. After being washed twice with RPMI, cells (1×10^7^) were either infected with PR8 (5×10^6^ pfu/3 ml) virus or mock infected for 30 min at room temperature. Cells were washed in RPMI twice and cultured for 5 days in a 1∶1 mixture of PBS and RPMI containing 10% FBS in culture dishes (Nunc, Roskilde, Denmark). In some groups, we used BALF from infected WT mice for stimulation. To inhibit IFN-I signaling, we used anti-IFNAR1 blocking antibody (100 ng/ml; MAR1-5A3; BioLegend, San Diego, CA).

### Microarray analysis

Monocytes were sorted from lung of WT and *Ifnar1* ^−/−^ mice at 5 dpi. RNA from each cell subset was extracted by RNA Isolation Kit (Qiagen, Valencia, CA). cDNA microarray analysis was performed using a MouseRef-8 v2 Expression Beadchip Kit (Illumina, Inc., San Diego, CA).

### Statistics

We used a paired two-sample *t*-test for analysis, except for survival data for which we used Kaplan-Meier analysis. *p<0.05, **p<0.01, and ***p<0.001 were considered significant.

## Supporting Information

Figure S1 Characterization of BALF cells in influenza-infected mice. (A) WT Ly6C^hi^ monocytes do not suppress CD4^+^ T cell proliferation. Naïve splenic CD4^+^ T cells were sorted by MACS and CFSE labeled. 5×10^4^ cells were plated on microwells coated with CD3 (10 µg/ml) and CD28 (1 µg/ml) mAbs. Ly6C^hi^ monocytes were FACS sorted from infected lung of WT mice at 3 dpi. Indicated ratios of monocytes and CD4^+^ T cells were co-cultured for 3 days. (B) Surface expression of co-stimulatory molecules on sub-populations in influenza-infected BALF at 5 dpi. AM, alveolar macrophages; N, neutrophils; DP, double-positive cells; Mo, monocytes. (C) Surface expression of various markers related to macrophages, dendritic cells, lymphocytes, and NK cells on monocytes in influenza infected lung.
(0.37 MB PDF)

Figure S2 Neutrophil and Ly6C^hi^ monocyte recruitment in different experimental models. PR8 virus (1×10^5^ pfu) was challenged in different mouse strains. (A) CD11c^neg^ cells were then analyzed using Ly6C- and Ly6G-specific Abs. TLR (*Myd88^−/−^Trif^−/−^*) and RLR (*Ips-1^−/−^*) signaling-deficient mice were able to generate Ly6C^hi^ monocytes. (B) *Ips-1^+/+^* and *Ips-1^−/−^* mice were infected with 1×105 pfu of PR8 and IFN-α production was determined in BALF at 3 dpi (*n* = 3).
(0.13 MB PDF)

Figure S3 Passively transferred activated neutrophils induce inflammation in the lung. (A) Schematic diagram of experimental procedure. Donor mice were infected with 1×10^5^ pfu of PR8 virus and sacrificed at 5 dpi. Neutrophils were sorted and passively transferred to recipient mice intranasally (1×10^6^ cell/mice). (B) H&E staining of lung from recipient mice at 2 days after transfer. (C) Cytokine expression in the BALF of recipient mice at 5 days after transfer.
(0.66 MB PDF)

Figure S4 Comparison of splenic monocytes in WT and *Ifnar1* ^−/−^ mice. (A) Gating performed for monocyte analysis. “Lin” including B220, Ly6G, CD4, CD8, CD49b mAbs and “Mac/DC” including F4/80, CD11c, MHCII mAbs. (B) Comparison of Ly6C MFI among Ly6C^hi^ splenic monocytes from WT and *Ifnar1^−/−^* mice. (C) Numbers of neutrophils and Ly6C^hi^ monocytes in the spleen of naïve and infected mice. WT and *Ifnar1^−/−^* mice were infected with 1×10^5^ pfu of PR8 virus and compared with naïve mice at 36 h after infection. *p<0.05, and #p = 0.07.
(0.17 MB PDF)
