# Supplementary figures and images for: Type I Interferon Signaling Regulates Ly6Chi Monocytes and Neutrophils during Acute Viral Pneumonia in Mice
Source: PLoS Pathog. 2011 Feb 24;7(2):e1001304. doi: 10.1371/journal.ppat.1001304 (PMC3044702; doi:10.1371/journal.ppat.1001304)

**A**Ly6C<sup>hi</sup> Mo : CD4 T cells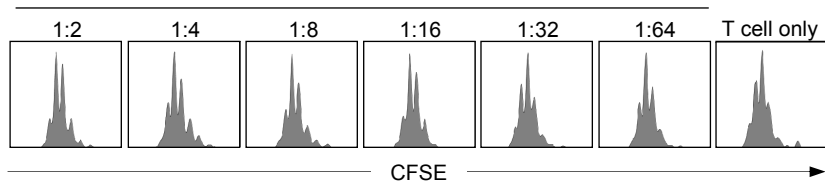**B**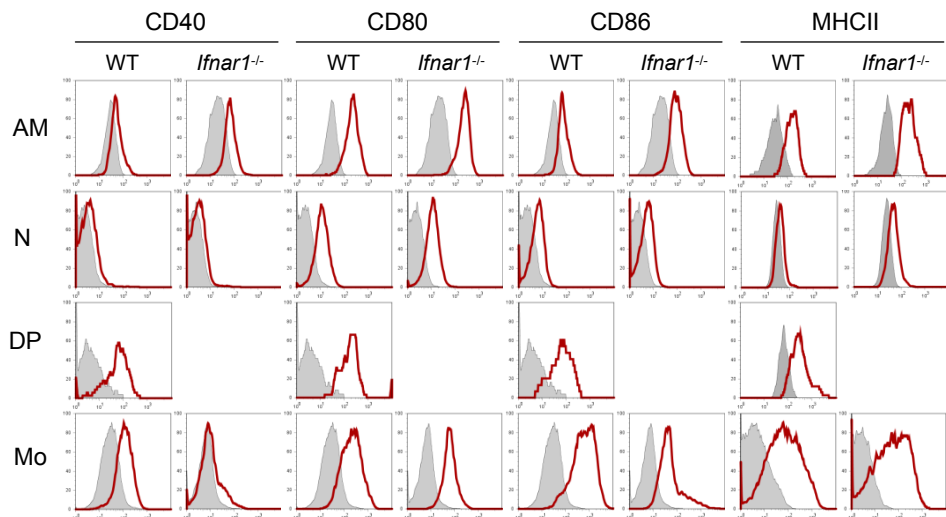**C**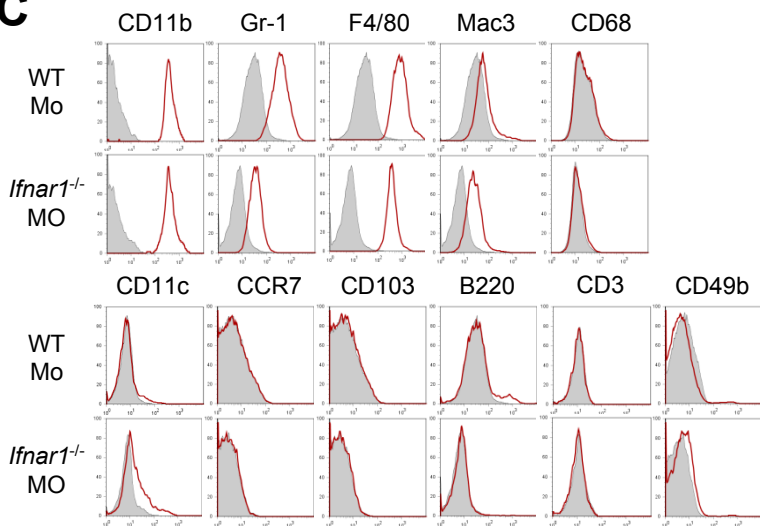

Supplement: Figure S1 — Characterization of BALF cells in influenza-infected mice. (A) WT Ly6Chi monocytes do not suppress CD4+ T cell proliferation. Naïve splenic CD4+ T cells were sorted by MACS and CFSE labeled. 5×10^4^ cells were plated on microwells coated with CD3 (10 µg/ml) and CD28 (1 µg/ml) mAbs. Ly6Chi monocytes were FACS sorted from infected lung of WT mice at 3 dpi. Indicated ratios of monocytes and CD4+ T cells were co-cultured for 3 days. (B) Surface expression of co-stimulatory molecules on sub-populations in influenza-infected BALF at 5 dpi. AM, alveolar macrophages; N, neutrophils; DP, double-positive cells; Mo, monocytes. (C) Surface expression of various markers related to macrophages, dendritic cells, lymphocytes, and NK cells on monocytes in influenza infected lung. (0.37 MB PDF) [file ppat.1001304.s001.pdf]

**A**CD11c<sup>neg</sup> gated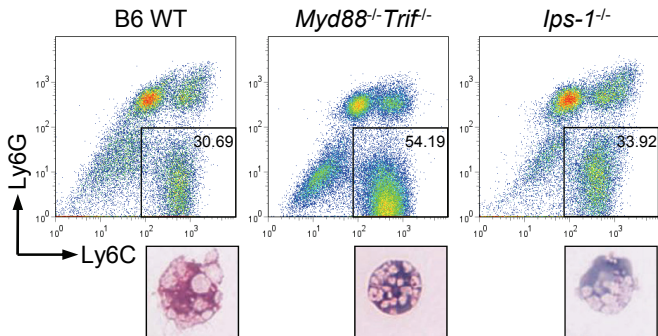**B**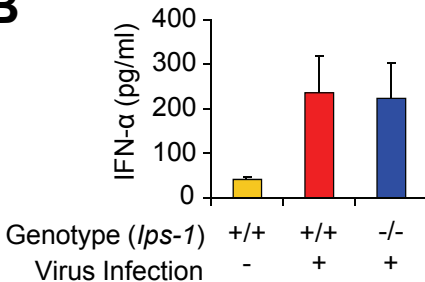

Supplement: Figure S2 — Neutrophil and Ly6Chi monocyte recruitment in different experimental models. PR8 virus (1×10^5^ pfu) was challenged in different mouse strains. (A) CD11cneg cells were then analyzed using Ly6C- and Ly6G-specific Abs. TLR (Myd88−/−Trif−/−) and RLR (Ips-1−/−) signaling-deficient mice were able to generate Ly6Chi monocytes. (B) Ips-1+/+ and Ips-1−/− mice were infected with 1×10^5^ pfu of PR8 and IFN-α production was determined in BALF at 3 dpi (n = 3). (0.13 MB PDF) [file ppat.1001304.s002.pdf]

**A**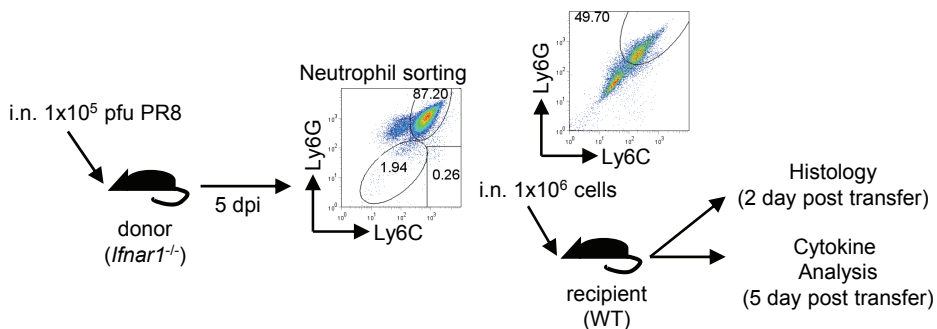**B**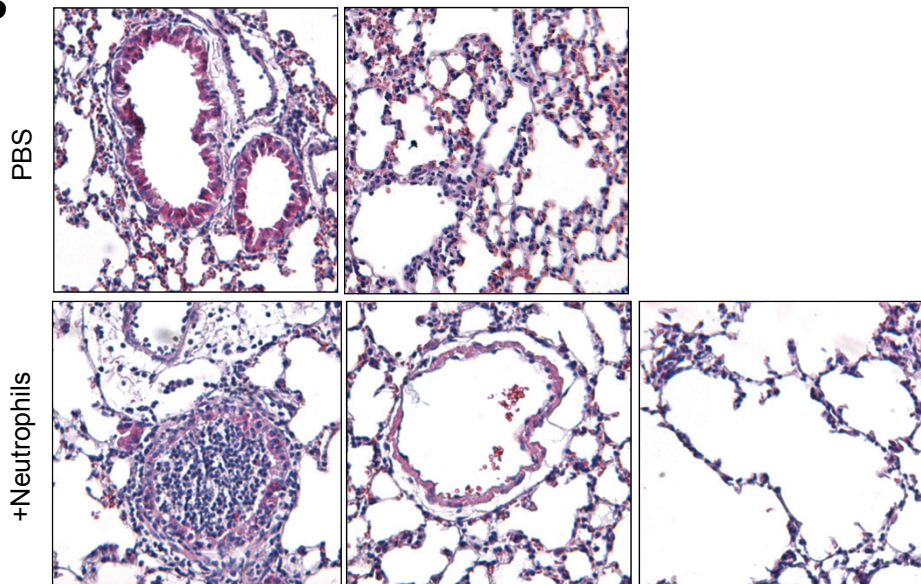**C**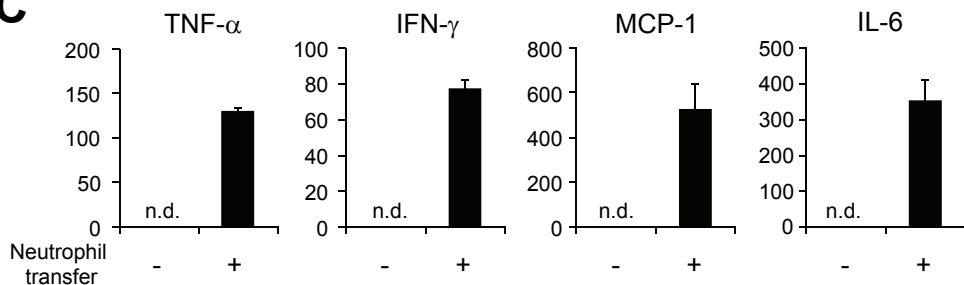

Supplement: Figure S3 — Passively transferred activated neutrophils induce inflammation in the lung. (A) Schematic diagram of experimental procedure. Donor mice were infected with 1×10^5^ pfu of PR8 virus and sacrificed at 5 dpi. Neutrophils were sorted and passively transferred to recipient mice intranasally (1×10^6^ cell/mice). (B) H&E staining of lung from recipient mice at 2 days after transfer. (C) Cytokine expression in the BALF of recipient mice at 5 days after transfer. (0.66 MB PDF) [file ppat.1001304.s003.pdf]

**A**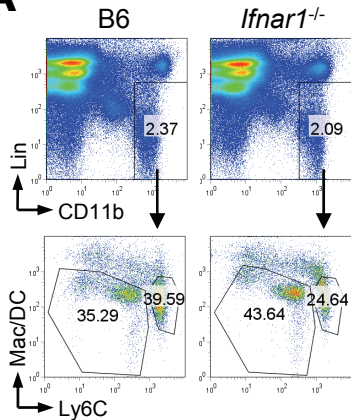**B**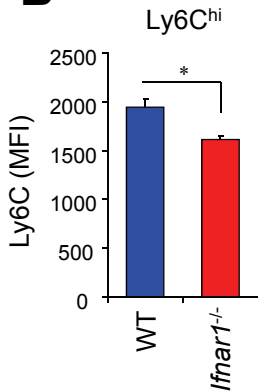**C**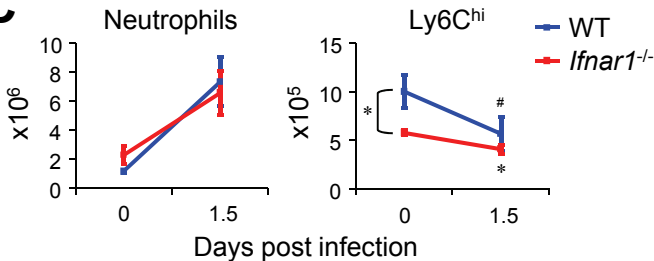

Supplement: Figure S4 — Comparison of splenic monocytes in WT and Ifnar1 −/− mice. (A) Gating performed for monocyte analysis. “Lin” including B220, Ly6G, CD4, CD8, CD49b mAbs and “Mac/DC” including F4/80, CD11c, MHCII mAbs. (B) Comparison of Ly6C MFI among Ly6Chi splenic monocytes from WT and Ifnar1−/− mice. (C) Numbers of neutrophils and Ly6Chi monocytes in the spleen of naïve and infected mice. WT and Ifnar1−/− mice were infected with 1×10^5^ pfu of PR8 virus and compared with naïve mice at 36 h after infection. *p<0.05, and #p = 0.07. (0.17 MB PDF) [file ppat.1001304.s004.pdf]
